# Assessment of Fungal Lytic Enzymatic Extracts Produced Under Submerged Fermentation as Enhancers of Entomopathogens’ Biological Activity

**DOI:** 10.1007/s00284-024-03702-z

**Published:** 2024-06-09

**Authors:** Cindy Mejía, Eddy J. Bautista, Lorena García, Juan Carlos Barrios Murcia, Gloria Barrera

**Affiliations:** 1 Centro de Investigación Tibaitatá, Corporación Colombiana de Investigación Agropecuaria - AGROSAVIA, Km 14 vía Mosquera - Bogotá, Cundinamarca, Colombia; 2 Corporación Colombiana de Investigación Agropecuaria - AGROSAVIA, Sede Central, Km 14 Vía Mosquera - Bogotá, Cundinamarca, Colombia

## Abstract

The application of enzymes in agricultural fields has been little explored. One potential application of fungal lytic enzymes (chitinases, lipases, and proteases) is as an additive to current biopesticides to increase their efficacy and reduce the time of mortality. For this, a screening of lytic overproducer fungi under submerged fermentation with a chemical-defined medium was performed. Then, the enzymatic crude extract (ECE) was concentrated and partially characterized. This characterization consisted of measuring the enzymatic activity (lipase, protease and, chitinase) and determining the enzyme stability after storage at temperatures of − 80, − 20 and, 4 °C. And lastly, the application of these concentrated enzymatic crude extracts (C-ECE) as an enhancer of spores-based fungal biopesticide was proven. *Beauveria* were not as good producers of lytic enzymes as the strains from *Trichoderma* and *Metarhizium.* The isolate *M. robertsii* Mt015 was selected for the co-production of chitinases and proteases; and the isolate *T. harzianum* Th180 for co-production of chitinases, lipases, and proteases. The C-ECE of Mt015 had a protease activity of 18.6 ± 1.1 U ml^−1^, chitinase activity of 0.28 ± 0.01 U ml^−1^, and no lipase activity. Meanwhile, the C-ECE of Th180 reached a chitinase activity of 0.75 U ml^−1^, lipase activity of 0.32 U ml^−1^, and protease activity of 0.24 U ml^−1^. Finally, an enhancing effect of the enzymatic extracts of *M. robertsii (*66.7%) and *T. harzianum* (43.5%) on the efficacy of *B. bassiana* Bv064 against *Diatraea saccharalis* larvae was observed. This work demonstrates the non-species-specific enhancing effect of enzymatic extracts on the insecticidal activity of conidial-based biopesticides, which constitutes a contribution to the improvement of biological control agents’ performance.

**Supplementary Information:**

The online version contains supplementary material available at 10.1007/s00284-024-03702-z.

## Introduction

Fungal enzymes have been exploited for several biotechnological applications, from the detergent, food, and textile industries to environmental approaches through bioremediation and transformation of wastes, and even therapeutic purposes (biomedicine) [1, 2]. Currently, the enzyme source production is dominated by filamentous fungi (60%), bacteria (24%), yeasts (4%), plants, and animals (10%), and it is expected that the industrial enzyme market will grow at a compound annual (CAGR) rate of 6.6% from 2023 to 2028 [2–4]. The success of microbial enzymes lies in their pH tolerance, thermostability, high yield, low operational cost, and easy and cheap downstream processing [1, 5]. Nowadays, new research has focused on exploring new sources of enzymes from different fungal isolates such as endophytes and entomopathogens [5]. The main enzymes with potential agriculture applications include lipases, chitinases, cellulases, proteases, xylanases, and pectinases to transform recalcitrant agricultural wastes (lignocellulose residues) into value-added products, increase soil fertility, and potentiate the biological control (biopesticides and biofungicides) [5, 6].

Entomopathogenic fungi such as *Beauveria* and *Metarhizium* are the most common biological control agents characterized to control different insect pests worldwide [7]. Recently, *Trichoderma* known as a biological control agent of soil-borne diseases has been also described as an entomopathogenic fungi [8]. The main *Trichoderma* mechanisms of action are parasitism, repellent activity, production of antifeeding compounds, secondary metabolites and enzymes against different insect orders [8, 9]. The capability of entomopathogens to colonize and control insect pests is driven mainly by lytic enzymes [10, 11]. First, lipases degrade the outer cuticle, increasing the adhesion of the fungi spores to insect cuticles by increasing the hydrophobic interactions between the fungus and the cuticle surface. Then, the proteases break down the peptide bonds of the sclerostin proteins from the exocuticle, revealing the chitin fibers. Finally, the exochitinases and endochitinases work together to break the chitin fibers [12]. However, the characterization of these enzymes has focused mainly on the fungi-cuticle insect interaction and selection of most virulent isolates [11, 13], but the use of enzymatic extracts as enhancers of biological control agents has been less explored [6].

The production of fungal extracellular lytic enzymes has been conducted through solid-state fermentation (SSF) or submerged fermentation (SmF). Nonetheless, SmF commonly uses synthetic or standardized media that allow increased control of the components [14]. SmF is mainly used in the production of enzymes and secondary metabolites secreted into the broth due to easy parameter control, large volume processing, reduced fermentation time, low labor intensity, easy recovery of products and purification steps and good technological basis for scaling to industrial level compared to SSF [15, 16]. Crude lytic enzymatic extracts produced through SmF of entomopathogenic fungi such as *Beauveria*, *Metarhizium,* and *Isaria* have shown great potential to control insect pests [17, 18].

Biopesticides are naturally occurring compounds that are used to control different agricultural pests [19]. Currently, most biopesticides registered in Colombia are based only on spores, conidia, or biomass of microorganisms. However, these types of biopesticides still have problems such as slow action and variations in efficacy in the field mainly due to the susceptibility of microorganisms to environmental conditions such as solar radiation, temperature fluctuations, and desiccation [20]. Consequently, biopesticides are applied frequently combined with chemical pesticides to increase crop protection [6], but these products are hazardous to human health and the environment, and in some cases are not compatible with the microorganisms [6, 21]. To overcome this problem, the use of enzymes combined with fungal biopesticides has gained interest as an eco-friendly alternative to enhance the insecticidal activity [22, 23]. Thus, the use of enzymes could diminish the field doses of biopesticides and increase their effectivity and, in consequence, reduce chemical pesticides applications. The potential use of the enzymes opens a new approach to *green technology* for the development of more sustainable and efficient biopesticides which may be the future of sustainable pest control worldwide [6, 24]. Therefore, we performed a screening of lytic overproducer fungi under SmF fermentation, then, the enzymatic crude extract was concentrated and partially characterized, and finally, the application of these concentrated enzymatic crude extracts as an enhancer of spores-based fungal biopesticide was proven.

## Materials and Methods

### Microorganisms and Inoculum Preparation

Nine strains were obtained from the Bank of Microorganism for Biological Control of AGROSAVIA to screen for lytic enzyme overproducers. They were: *Beauveria bassiana* encoded as Bv060, Bv062, and Bv064; *Metarhizium anisopliae* Mt04, *Metarhizium robertsii* Mt015, and *Metarhizium brunneum* CA-3 and *Trichoderma koningiopsis* Th003, *Trichoderma harzianum* Th180, and *Trichoderma virens* Gl006. These strains had previously shown biocontrol activity against *Cerotoma tingomariana, Diatraea saccharalis, Rhammatocerus schistocercoides, Premnotrypes vorax* and *Gonipterus* sp., and phytopathogens such as *Fusarium oxysporum*. These are native fungi from Colombia and their use is granted by Agreement to Access Genetic Resources and its Derived Products No. 168 of 2017 between Agrosavia and Colombia´s Ministry of Environment and Sustainable Development.

The fungi were grown on potato dextrose agar (PDA) and incubated at 25°C for seven days. Inoculum was prepared by aseptically scraping off each Petri dish into sterile 0.1% Tween 80 and vigorously shaking to obtain a conidial suspension. Concentrations were adjusted to 1×10^6^ conidia ml^−1^ by hemocytometer.

### Entomopathogenic Fungi Screening and Selection for Lytic Enzyme Production Under Submerged Fermentation

The nine strains were grown under submerged fermentation SmF process to quantify their lytic enzyme production. The SmF was carried out following the methodology described by Dhawan and Joshi, 2017 using a culture medium with inducers for lipases, proteases, and chitinases production [25]. The culture medium had the following composition (g l^−1^): KH_2_PO_4_, 3.0; K_2_HPO_4_, 1.0; MgSO_4_, 0.7; (NH_4_)_2_SO_4_, 1.4; NaCl, 0.5; CaCl_2_, 0.5: yeast extract, 0.5; peptone, 0.5; chitinase, 5.0; and olive oil (commercial brand YBARRA S.A.), 5 ml l^−1^, pH 5.6. The SmF was carried out on a 125 ml flask with a liquid-to-headspace ratio of 1:5. The culture medium was inoculated with the conidial suspension of each microorganism and incubated at 28 °C with constant agitation of 150 rpm for 14 days. Three biological replicates were conducted for each strain. Samples were withdrawn at 10 days and 14 days. The samples were centrifuged at 10,000 rpm for 10 min, and the supernatant was recovered and used as the enzymatic crude extract (ECE) for the enzyme tests.

### Enzymatic Activity Assays

#### Lipase Activity

Lipase activity was measured in the ECE and C-ECE following the methodology described by Beys da Silva et al. [26] and Glogauer et al. [27]. Briefly, 20 µl of ECE or C-ECE were mixed with 230 µl of the substrate. The substrate was prepared with 3 mg of *p*-nitrophenyl palmitate (*p*NPP) in 1 ml of isopropanol and 9 ml of 50 mM Tris–HCl pH 8 (Arabic gum 1 mg ml^−1^, Triton X-100 4 mg ml^−1^). The reaction was incubated at 37 ± 2 °C for 30 min. The absorbance of *p*-nitrophenol production was determined at 410 nm in a spectrophotometer [26, 27]. One unit of lipase activity was defined as the amount of enzyme that released 1 μmol p-nitrophenol per minute at the conditions of the assay. The assays were run in triplicates.

#### Chitinase Activity (N-Acetylglucosaminidase)

Chitinase activity was measured in the ECE and C-ECE by following the procedure previously described [23]. Thus, 20 μl of sample were mixed with 100 μl of *p*-nitrophenyl-N-acetyl-β-D-glucosaminide (*p*NP‐GlcNAc) (1 mg ml^−1^ in citrate buffer 0.1 M pH 5). The reaction was incubated at 37 ± 2 °C for 30 min and stopped with 150 μl NaOH-glycine pH 10.4. The absorbance was measured at 400 nm and the concentration of p-nitrophenol was estimated. One unit of the enzyme was defined as the amount of enzyme that released 1 μmol of p-nitrophenol per minute at the conditions of the assay. The assays were run in triplicates.

#### Protease Activity

The protocol used to quantify protease activity was previously described by Cupp-Enyard [28]. Thus, 25 µl of ECE or C-ECE were mixed with 130 µl of substrate (0.65% casein in buffer 50 mM Tris-HCl pH 7.5). The reaction was incubated at 37 ± 2 °C for 10 min. Then, 130 µl of 110 mM trichloroacetic acid were added to the mixture and were incubated at 37 °C for an additional 20 min. Later, the samples were centrifuged at 12,000 rpm for 10 min, and 50 µl were withdrawn, and 125 µl of 500 mM sodium carbonate were added, followed by 25 µl of Folin-Ciocalteu’s reagent. The reaction was incubated at 37 °C for 30 min. Finally, the absorbance was measured at 660 nm and the concentration of tyrosine was estimated. One unit of the enzyme was defined as the amount of enzyme that released 1 μmoL of tyrosine per minute at the conditions of the assay. The assays were run in triplicates.

### Enzyme Kinetics

After the initial screening of the nine strains under submerged fermentation, we selected the highest enzyme-producing fungi and, the enzyme production kinetic experiment was carried out in the same culture medium and same conditions as described in “Entomopathogenic Fungi Screening and Selection for Lytic Enzyme Production Under Submerged Fermentation” section. Samples were withdrawn every two days for 14 days for enzymatic activity quantification. Enzyme quantification was performed using the methodologies previously described in “Enzymatic Activity Assays” section.

### Concentration of Enzymatic Crude Extract

After 14 days of fermentation of *M. robertsii* Mt015 and *T. harzianum* Th180*,* 50 ml of culture media were taken from each fermentation and spun for 10 min at 4 °C at 10,000 rpm. Then, the supernatant was used as ECE and dialyzed with a membrane (molecular weight cut-off of 12–14 KDa) in Polyethylene glycol 20,000 to get the concentrated enzymatic crude extract (C-ECE) which was used in the shelf life experiments and bioassays.

### Enzyme Shelf Life

The C-ECE from *M. robertsii* Mt015 and *T. harzianum* Th180 were stored at different temperatures − 80 °C, − 20 °C, 4 °C, and 15 °C for 30 days with sampling at 7th and 30th days for enzymatic activity quantification. The responses were expressed as relative activity between initial (${U}_{0}$) and final (${U}_{f}$) enzymatic activities, Eq. 1.

1 $$\frac{{U_{f} }}{{U_{0} }} \times 100\%$$

### C-ECE as Enhancer of Biological Activity of *B. bassiana* Bv064 Against* Diatraea saccharalis*

#### *B. bassiana* Bv064 Conidial Production

Conidia of *B. bassiana* Bv064 were produced in a semi-solid fermentation process [29]. Aluminum trays with 10% w/v rice powder were previously sterilized at 120 °C for 20 min and cooled at room temperature before inoculation. Each tray was inoculated with 2 ml of conidia suspension containing 1 × 10^6^ con ml^−1^. Trays were covered with a translucent plastic film and incubated at 25 °C for 7 days. Then, the plastic film was replaced with a paper towel to promote sporulation and drying of the substrate and conidia. Conidia were harvested by sweeping the culture medium surface with a brush and sieved through a 150 μm mesh. Dry conidia were used to prepare a conidial suspension of 1 × 10^5^ con ml^−1^ (lethal media concentration LC_50_) for the following experiments.

#### Effect of the C-ECE Over *B. bassiana* Bv064 Germination

The effect of C-ECE of Mt015 and C-ECE of Th180 over *B. bassiana* Bv064 conidia germination was evaluated following a methodology previously reported [23]. The C-ECE of Mt015 had a protease activity of 12 U ml^−1^, chitinase activity of 0.18 U ml^−1^, and the C-ECE of Th180 had a chitinase activity of 0.75 U ml^−1^, lipase activity of 0.32 U ml^−1^, and proteases activity of 0.24 U ml^−1^. Nonetheless, from this point forward the C-ECE for each fungus will be referred to only using the major enzyme. The treatments were: (1) A mixture of C-ECE Th180 with Bv064 conidia suspension (1 × 10^6^ con ml^−1^); (2) A mixture of C-ECE Mt015 with Bv064 conidia suspension; And (3) control treatment, Bv064 conidia suspension (1 × 10^6^ con ml^−1^), without addition of C-ECE. Treatments were incubated at 25 °C for 1 h. Germination was evaluated following the method reported by Santos et al. [30]. Samples (100 μl) of each treatment were inoculated on plates with malt extract agar (0.1% w v^−1^) supplemented with chloramphenicol (0.1% w v^−1^) and benomyl (0.00005% w v^−1^). Petri dishes were incubated at 25 °C and the percentage of germinated conidia was evaluated under light microscopy at 24 h. At the same time, the germ tubes length of 300 conidia was measured for each treatment. The photos were taken with a microscope Axio Lab. A1 Zeiss at 40x, and a camera Axiocam Erc 5 s Zeiss, and the measurement of germ tube length was performed with program ZEN 2.3 (Carl Zeiss Microscopy GmbH, 2011).

#### Bioassay of *B. bassiana–D. saccharalis*

The bioassay was carried out with *B. bassiana* Bv064 against *D. saccharalis* which had previously been standardized [23] to determine the effect as an enhancer of the enzyme over the biocontrol agent. The dorsa of *D. saccharalis* second instar larvae were inoculated by applying 2 μl of each treatment (Table 1). Inoculated larvae were individually transferred to 0.5 oz capacity plastic cups containing one fresh grain of maize as a feeding substrate. The cups were incubated at 25 °C (60% RH). Larval mortality was recorded at 2, 4, 6, 8, 10, 12, and 14 days. The experimental unit consisted of 15 larvae with three biological replicates (45 larvae) per treatment. Efficacy (treatment mortality corrected with mortality in the control treatment) was calculated using the Schneider–Orelli formula [31].

**Table 1:** Bioassay’s treatments of C-ECE Mt015/ C-ECE Th180 mixed with *B. bassiana* Bv064 conidia against *D. saccharalis* second instar larvae under laboratory conditions

Treatment	C-ECE *M. robertsii* Mt015	C-ECE *T. harzianum* Th180
T1	Control (0.1% Tween 80)	Control (0.1% Tween 80)
T2	Sterile culture medium	Sterile culture medium
T3	Bv064 CL_50_ (1 × 10^5^ con ml^−1^)	Bv064 CL_50_ (1 × 10^5^ con ml^−1^)
T4	C-ECE (proteases 4.0 U ml^−1^)	C-ECE (chitinases 0.2 U ml^−1^)
T5	C-ECE (proteases 8.0 U ml^−1^)	C-ECE (chitinases 0.4 U ml^−1^)
T6	C-ECE (proteases 12 U ml^−1^)	C-ECE (chitinases 0.8 U ml^−1^)
T7	Bv064 + C-ECE (proteases 4.0 U ml^−1^)	Bv064 + C-ECE (chitinases 0.2 U ml^−1^)
T8	Bv064 + C-ECE (proteases 8.0 U ml^−1^)	Bv064 + C-ECE (chitinases 0.4 U ml^−1^)
T9	Bv064 + C-ECE (proteases 12 U ml^−1^)	Bv064 + C-ECE (chitinases 0.8 U ml^−1^)

A parallel bioassay was conducted to confirm that the enhancing effect is caused mainly by the enzymes present in the crude extract of *M. robertsii* Mt015 and *T. harzianum* Th180*.* Briefly, the C-ECEs were adjusted to the highest enzymatic activities tested (C-ECE Mt015—proteases 12 U ml-1 and C-ECE Th180—chitinases 0.8 U ml-1). The C-ECEs were incubated at temperature of 100 °C for 1 h to inactivate the enzymes. After heating, the C-ECEs were allowed to cool in room temperature. Later, the protease and chitinase activity was measured in both extracts to confirm inactivation of the enzymes. *D. saccharalis* larva were inoculated with the mixture of heat-treated ECEs with *B. bassiana* Bv064 conidia.

### Statistical Analysis

All data from screening, enzyme production, germination and bioassay were checked for normality and homogeneity variance using Shapiro–Wilk and Bartlett tests, respectively. Data were analyzed by one-way ANOVA and significant differences were determined with Tukey test (95% confidence) using Statistix software (Version 7.0 Analytical Software, Florida, U.S.A.). A pairwise statistical comparison test was carried out between the germ tube size data for the different treatments using a Games-Howell´s test, with the R´ggstatsplot´ package [32] with a confidence level of 95%. The *p*-values for comparisons involving more than two groups were adjusted through Holm’s multiple comparison correction. All figures were created with the R ´ggplot2´ package [33].

## Results

### Entomopathogenic Fungi Screening and Selection for Lytic Enzyme Production Under a SmF Process

**Fig. 1:**
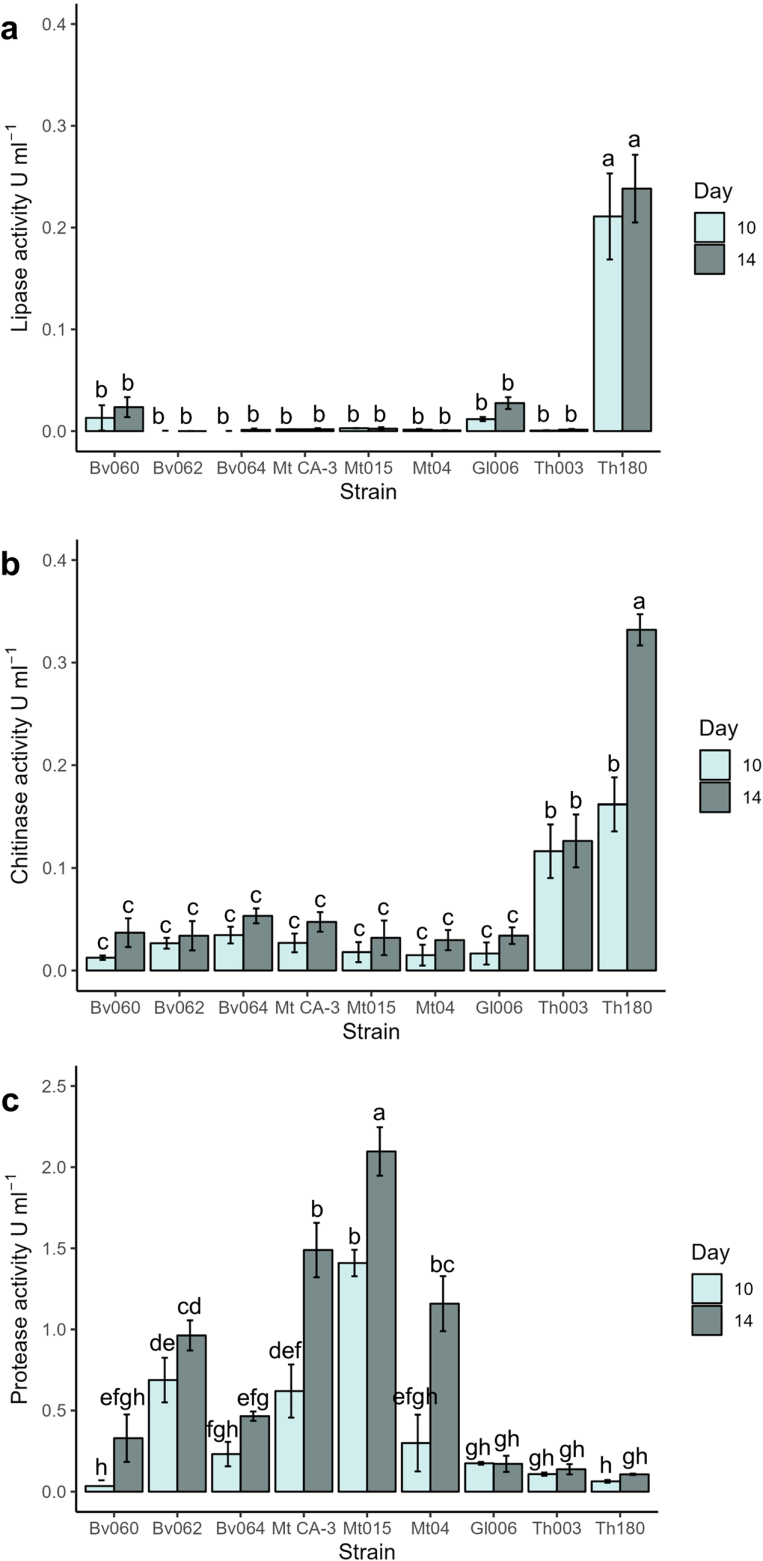
Lytic enzymatic activity for nine fungi strains (*B. bassiana* Bv060, *B. bassiana* Bv062, and *B. bassiana* Bv064; *M. anisopliae* Mt04, *M. robertsii* Mt015, and *M. brunneum* CA-3; and *T. koningiopsis* Th003, *T*. *harzianum* Th180, and *T. virens* Gl006) under SmF fermentation at different days. **a** Lipase activity, **b** Chitinase activity, **c** Protease activity. The values are the mean of the triplicate biological experiments. The error bars are the standard deviation. Values with the same letters do not have a significant difference by Tukey test (95%)

The capability of microorganisms to produce lipases, chitinases and proteases was assessed in submerged fermentation, and the best enzyme-producing microorganisms were selected. The nine strains were able to grow in the SmF fermentation with the co-production of lytic enzymes. Strains *T. koningiopsis* Th003, *B. bassiana* Bv062, *B. bassiana* Bv064, *M. anisopliae* Mt04, *M. robertsii* Mt015, and *M. brunneum* CA-3 were unable to produce lipases under the conditions of the experiment (Fig. 1a). Meanwhile, *T. harzianum* Th180 reached the highest lipase value of 0.24 ± 0.03 U ml^−1^ at day 14 (*F* = 57.8, gl = 53, *p* < 0.05) (Fig. 1a). This strain also had higher chitinase activity 0.33 ± 0.02 U ml^−1^, followed by *T. koningiopsis* Th003 with 0.13 ± 0.02 U ml^−1^. Meanwhile, the strains from the genera *Beauveria* and *Metarhizium* had the lowest values of chitinase activity between 0.03 and 0.05 U ml^−1^ (*F* = 57.5, gl = 53, *p* < 0.05) (Fig. 1b). In contrast, the three strains from the genera *Metarhizium* showed the highest protease activity (Fig. 1c), *M. robertsii* Mt015 showed the highest value, 2.1 ± 0.2 U ml^−1^, at day 14, followed by *M. anisopliae* CA-3 with 1.5 ± 0.2 U ml^−1^ and *M. robertsii* Mt04 1.2 ± 0.2 U ml^−1^ (*F* = 64.2, gl = 53, *p* < 0.05). Meanwhile, Bv062 had the highest protease activity of the three *Beauveria* strains with 0.96 ± 0.09 U ml^−1^. Finally, the three strains from the genera *Trichoderma* showed the lowest levels of proteases between 0.11 and 0.17 U ml^−1^. Therefore, *T. harzianum* Th180 was chosen for the coproduction of lipases, chitinases and proteases, whereas *M. robertsii* Mt015 was chosen for protease and chitinase coproduction.

### Enzyme Coproduction Over Time Under SmF Process for *M. robertsii *Mt015 and *T. harzianum* Th180

**Fig. 2:**
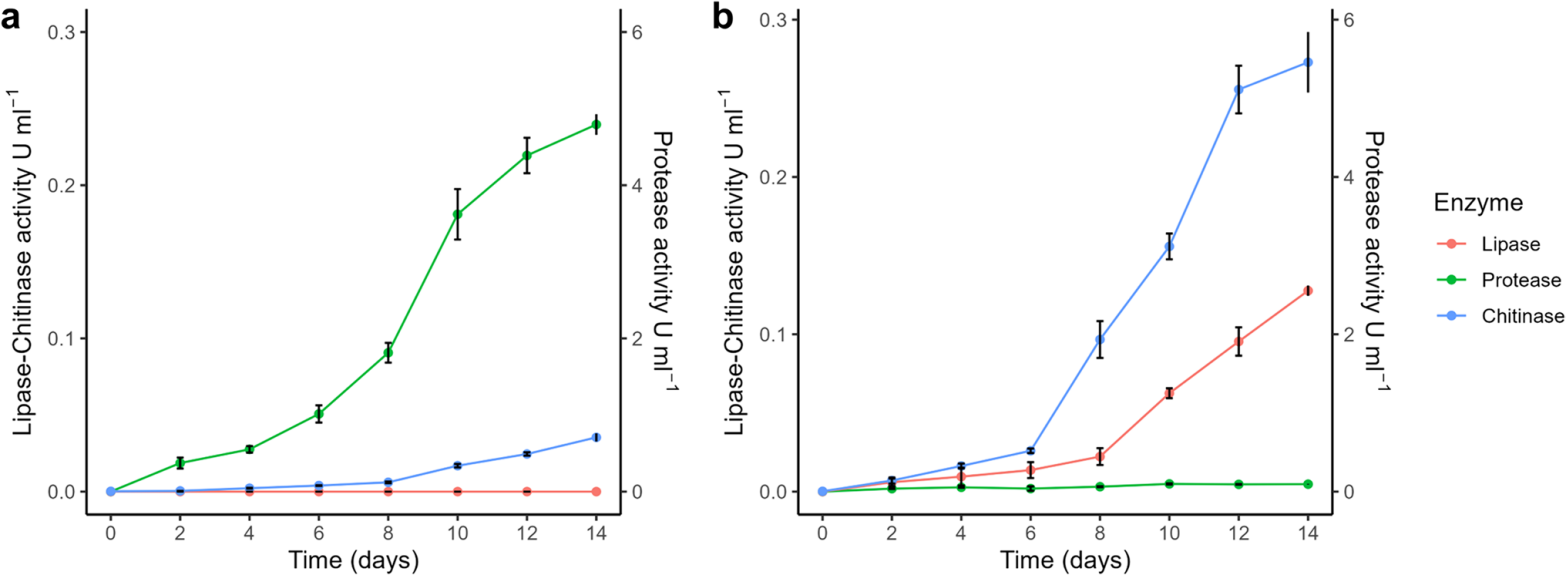
Enzymatic coproduction (protease, chitinase, and lipase) by **a** *M. robertsii* Mt015, **b** *T. harzianum* Th180 under SmF process. The values are the mean of the triplicate biological experiments. The error bars are the standard deviation

The enzyme co-production over time studies allowed determining the highest point of lytic enzyme´s co-production. The fungus *M. robertsii* Mt015 was outstanding for its protease production, with the highest value of 4.8 ± 0.1 U ml^−1^ after 14 days of SmF fermentation, which was a value statistically similar to the protease activity at days 10 and 12, as shown in Fig. 2a. Also, the fungus produced the highest chitinase activity at day 14 with 0.04 ± U ml^−1^, whereas no lipase production was detected at any time of the SmF fermentation.

On the other hand, *T. harzianum* Th180 co-produced mainly chitinases followed by lipases, and some proteases at the SmF fermentation process (Fig. 2b). Extracellular chitinases were detected starting on day 4 with an enzymatic activity below 0.1 U ml^−1^. Then, the production of chitinase increased with time reaching a value of 0.27 ± 0.02 U ml^−1^ on day 14. Lipase production had a similar behavior as chitinases with an increasing production over time to a maximum value of 0.12 ± 0.003 U ml^−1^ within 14 days of SmF fermentation. Some proteases were produced with the highest activity at day 10 with 0.10 ± 0.007 U ml^−1^.

### Concentration of Enzymatic Crude Extract

Dialysis of enzymes allowed concentrating the fungal ECE and removing some components such as salts, small organic molecules, and peptides of culture medium that could interfere with the enzymatic activity. Accordingly, the ECE from *M. robertsii* Mt015 was dialyzed against polyethylene glycol and volume was reduced tenfold to have a protease activity of 18.6 ± 1.1 U ml^−1^, chitinases activity of 0.28 ± 0.01 U ml^−1^ and no lipase activity (Supplementary figure S1). Also, the ECE of *T. harzianum* Th180 was dialyzed, and volume was reduced 7.5-fold reaching a chitinase activity of 0.75 ± 0.09 U ml^−1^, lipase activity of 0.32 ± 0.00 U ml^−1^, and a protease activity detected of 0.24 U ml^−1^ (Supplementary figure S1). Thus, the C-ECE of *M. robertsii* Mt015 had 77 times more protease activity and 2.7 times less chitinase activity than the C-ECE of *T. harzianum* Th180.

### Enzyme Shelf Life

**Fig. 3:**
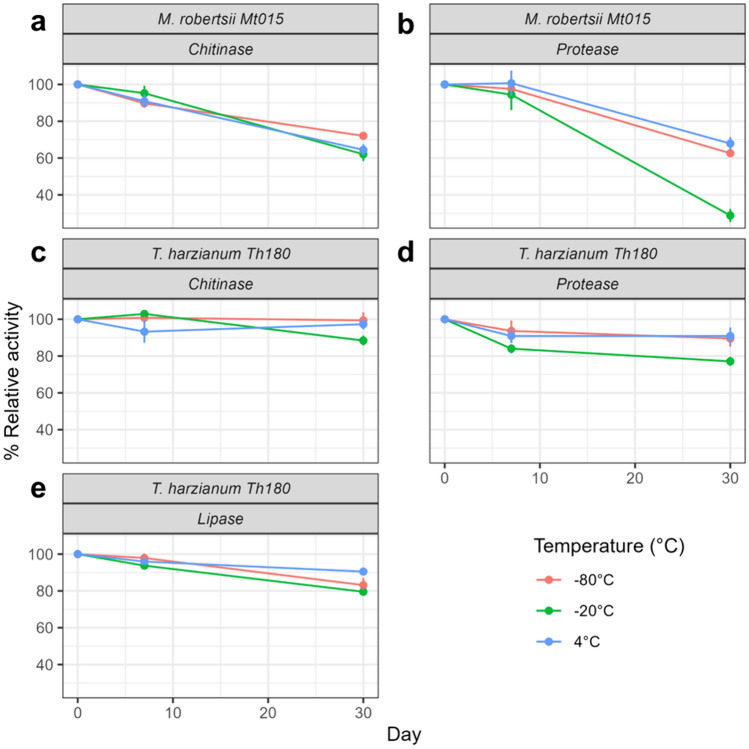
Relative enzymatic activity of C-ECE *M. robertsii* Mt015 **a** chitinase, **b** protease, and C-ECE *T. harzianum* Th180 **c** chitinase, **d** protease, **e** lipase for the three storage temperatures − 80 °C (red), − 20 °C (green), and 4 °C (blue) at days 7 and 30 (Color figure online)

The C-ECE of *M. robertsii* Mt015 had lower stability of chitinases compared to the C-ECE of *T. harzianum* Th180. The highest loss of activity of the C-ECE Mt015 was around 72% on day 30 of storage compared to 20% of the C-ECE Th180 on day 30. During the 30 days of storage, the chitinase activity decreased by 35.6% ± 2.5 at 4 °C, 37.9% ± 3.2 at − 20 °C, and 27.9% ± 0.9 at − 80 °C (Fig. 3a). Hence, the best temperature to keep the chitinase activity of C-ECE Mt015 was − 80 °C. Moreover, proteases from *M. robertsii* Mt015 were able to keep their relative activity within their first seven days of storage with values of 100.0% ± 3.0 at 4 °C, 94.4% ± 3.0 at − 20 °C, and 97.5 ± 1.0 at − 80 °C. However, 30 days of storage had a negative effect on the proteases with a relative enzymatic activity of 67.9% ± 3.0 at 4 °C, 28.8% ± 3.0 at − 20 °C, and 62.6% ± 0.9 at − 80 °C (Fig. 3b).

The best temperature to store the C-ECE Th180 to keep the relative chitinase activity for up to 30 days was − 80 °C at 99.4% ± 3.94, followed by 4 °C with 97.3% ± 2.3, and − 20 °C at 88.4% ± 2.2 (Fig. 3c). Further, the protease activity was more affected by the − 20 °C as temperature of storage with a relative protease activity of 77.1% ± 2,0 at 30 days as shown in Fig. 3d, similar behavior at this temperature was seen with the proteases from Mt015. Nonetheless, the higher decreases in protease activity at temperature − 80 °C and 4 °C was at the first 7 days of storage, then the relative protease activity was stable until the 30 days. Meanwhile, lipases were able to keep a high relative activity for the first 7 days at temperatures of 4 °C (96% ± 1.1) and − 80 °C (98% ± 1.2), but the relative activity decreased to 90.5% ± 0.9 at 4 °C and 83.2% ± 3.4 at − 80 °C within 30 days of storage. Finally, the decrease in lipase activity at − 20 °C was significant within 30 days (*F* = 40.3, gl = 20, *p* < 0,05) with a relative lipase activity of 79.5% ± 0.4 (Fig. 3e).

### C-ECE as Enhancers of Biological Activity of *B. bassiana* Bv064 Against* Diatraea saccharalis*

#### C-ECE of Mt005 and C-ECE of Th180´s Effect Over In Vitro Germination and Germ Tube Length of *B. bassiana* Bv064

**Fig. 4:**
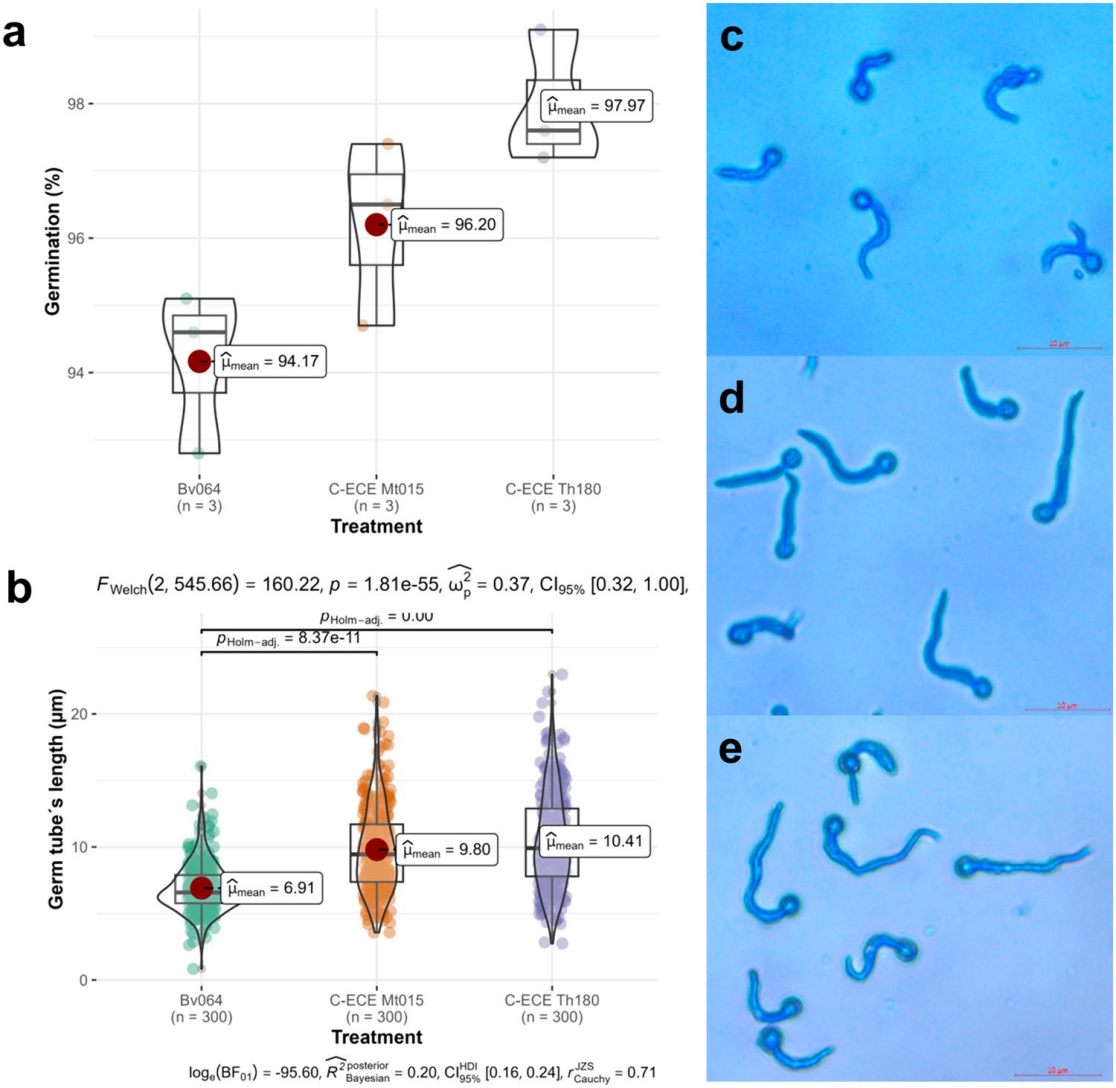
**a** In vitro germination (%) for *B. bassiana* Bv064´s conidia with and without the addition of C-ECE from *M. robertsii* Mt015 or *T. harzianum* Th180. Boxes denote data within 25th and 75th percentiles, and contain median (middle line) and mean (red dot) value notations; **b** Germ tube length (µm) from *B. bassiana* Bv064 with and without the addition of C-ECE from *M. robertsii* Mt015 and *T. harzianum* Th180. Boxes denote data within 25th and 75th percentiles, and contain median (middle line) and mean (red dot) value notations. *p-*values were calculated with the two-sided Holm-corrected Games-Howell´s test; **c** Photograph of conidia germination of *B. bassiana* Bv064; **d** Photograph of conidial germination of *B. bassiana* Bv064 with th addition of C-ECE from *M. robertseii* Mt015; **e** Photograph of conidial germination of *B. bassiana* Bv064 with addition of C-ECE from *T. harzianum* Th180

It is hypothesized that the addition of C-ECE of lytic enzyme to conidia of entomopathogen fungus will allow a fast spore germination and germ tube growth, which would result in a faster colonization of the pests [34]. Thus, the C-ECE of each fungus was mixed with *B. bassiana* Bv064´s conidia, and germination and germ tube length were measured. The addition of the C-ECE Th180 (chitinase activity of 0.8 U ml^−1^) to the conidia resulted in a statistically significant higher germination rate of 98% ± 1 compared to the conidia without any addition of enzymatic extract with 94.2% ± 1.2. Meanwhile, the C-ECE Mt015 (protease activity of 12 U ml^−1^) had a higher germination rate of 96.2% ± 1.4 than that of the control, but it was not statistically significant (Fig. 4a).

The germ tube’s length for the conidia under each treatment is presented in Fig. 4b, and a Games-Howell’s test was applied to the data. It showed a significantly higher mean of germ tube’s length on the conidia-treated with C-ECE from Mt015 (9.79 µm ± 3.29) or C-ECE from Th180 (10.41 µm ± 3.63) compared to the control mean (6.91 µm ± 1.89) (Fig. 4c–e). Only 6% of the *B. bassiana* Bv064 conidia sample had a germ tube´s length larger than 10 µm. On the other hand, when *B. bassiana* conidia were mixed with C-ECE from Mt015 or Th180, 42% and 49% of the conidia sample had germ tube’s length larger than 10 µm, respectively.

#### Bioassay

Every treatment with Bv064´s conidia showed signs of infection. Larva had pink coloration, necrotic tissue, and colonization and sporulation of the fungus were observed on the larva (Supplementary figure S2), whereas the treatments with only the C-ECE of any of the fungi did not show signs of infection.

**Fig. 5:**
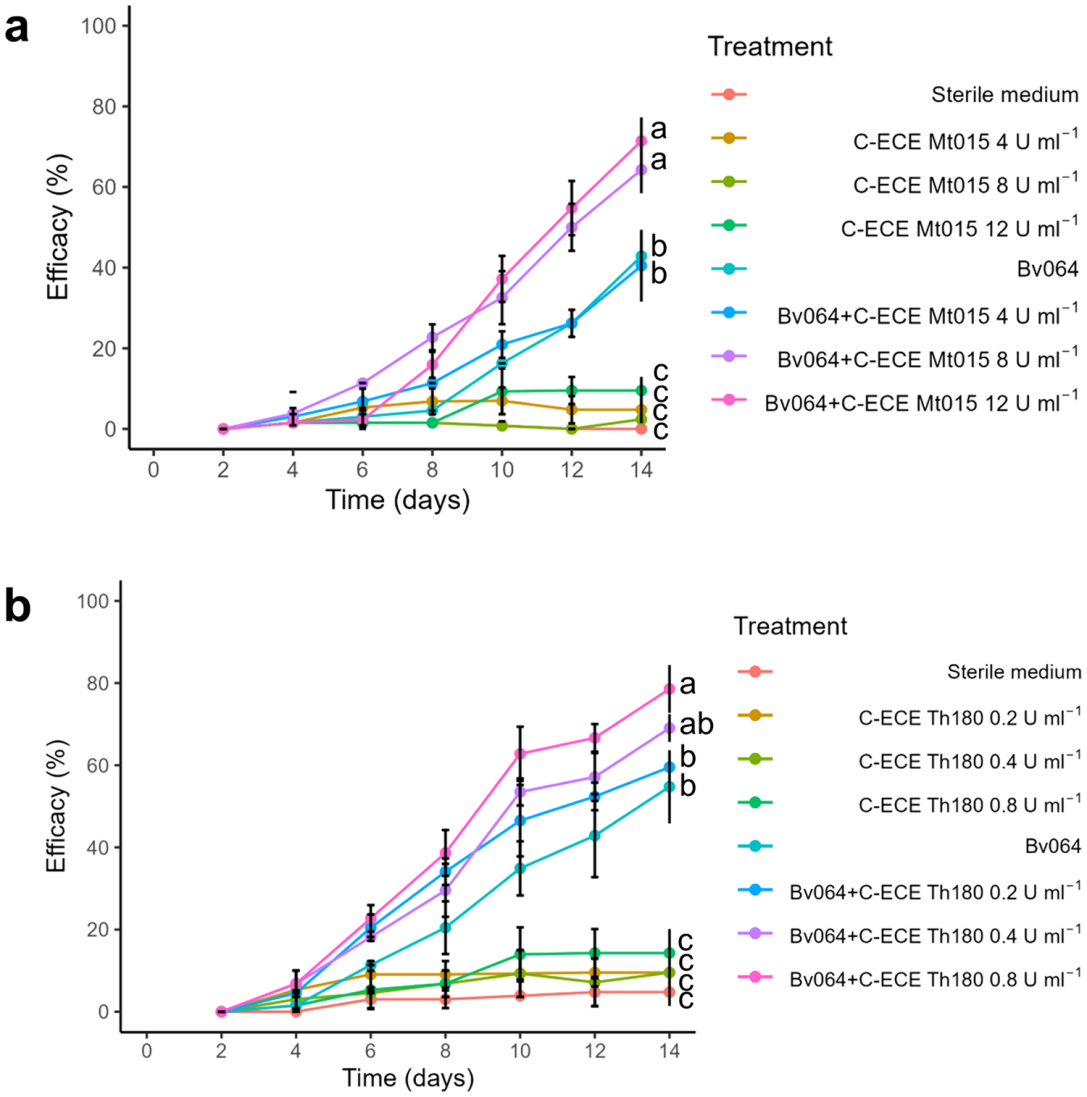
Efficacy of *B. bassiana* Bv064 conidia alone and mixed with concentrated enzymatic crude extracts (C-ECE) against *D. saccharalis* second instar larvae. **a** The C-ECE came from *M. robertsii* Mt015, the enzymatic units of each treatment correspond to proteases. **b** The C-ECE came from *T. harzianum* Th180, the enzymatic units of each treatment correspond to chitinases. Values with the same letters do not have a significant difference by Tukey test (95%)

The C-ECE of *M. robertsii* Mt015 had a significant effect on enhancing the insecticidal activity of Bv064’s conidia against *D. saccharalis* larvae. After day 10, the two highest concentrations of C-ECE (protease activity of 8 and 12 U ml^−1^) significantly increased the efficacy of Bv064´s conidia to 32.6 ± 6.6% and 37 ± 5.7%, respectively, compared to the Bv064´s conidia efficacy of 16.3 ± 0.0% (Fig. 5a). Whereas at day 14, the efficacy of the mixture C-ECE Mt015 (8 U ml^−1^ or 12 U ml^−1^) with Bv064´s conidia had an efficacy of 64.3 ± 5.8%, and 71.4 ± 5.8% compared to a Bv064’s conidia efficacy of 42.8 ± 5.8%, an increase of 50% and 66%, respectively (*F* = 62.5, gl = 23, *p* < 0.05).

Regarding the application of only the C-ECE of *T. harzianum* Th180 at the three chitinases enzymatic activities of 0.2, 0.4, and 0.8 U mL^−1^ on the *D. saccharalis*’s larva, the efficacy was below 15% compared to the other treatments except for the control (Fig. 5b). Meanwhile, a significant increase in efficacy was observed when the C-ECE of Th180 was added to the B. *bassiana* Bv064’s conidia compared to the conidia alone from day 6. The efficacy of Bv064’s conidia mixed with the C-ECE on day 8 was 34.1 ± 3.2% % (0.2 U ml^−1^), 29.6 ± 6.4%% (0.4 U ml^−1^), and 38.6 ± 5.6% (0.8 U ml^−1^) compared to the efficacy of the conidia alone of 20.5 ± 6.4%. Finally, at day 14, a statistically significant increase in efficacy was detected when the C-ECE at the higher concentration (chitinases of 0.8 U ml^−1^) was added to the Bv064’s conidia. The efficacy was 78.6 ± 5.8% (*F* = 74.4, gl = 23, *p* < 0.05), while the efficacy of the Bv064´s conidia alone was only 54.8 ± 8.9%, a 43% increase.

Also, the application of heat-treated C-ECEs of *M. robertsii* Mt015 and *T. harzianum* Th180 (Relative enzymatic activity of 0% after heating) mixed with Bv064’s conidia caused efficacies of 55.6 and 51.4% after 14 days of inoculation, respectively. These efficacies were similar to the efficacy of Bv064’s conidia alone, and lower than the efficacy of Bv064’s conidia mixed with non-heated C-ECEs. These findings demonstrate that the enhancing effect of the fungal crude extracts is caused mainly by the enzymes (data no showed).

## Discussion

The adoption and feasibility of biological control strategies depend on many critical points from the massive production process, shelf-life, storage conditions, and field application, but the efficacy and persistence of microorganisms (used as active ingredients of biopesticides) under field conditions are the most challenging ones. The use of chemical pesticides in crops is still the first option for pest management due to its effectiveness at relatively low costs, high residual effectiveness, higher yields of the crops, higher shelf life, and ease of storage, regardless of the harmful effect on the environment and its negatively impacts on human health [6]. To improve the performance of microbial pesticides, there are alternatives that include increasing virulence factors represented by lytic enzymes that support the insecticidal activity of microorganisms. These enzymes can be produced by recombinant technology using heterologous expression systems or produced under the fermentation process of entomopathogenic fungi [6]. For example, the application of crude extracts from the fermentation of *M. anisopliae*, *B. bassiana* and *I. fumosorosea* in adults of *Musca domestica* [18] or supernatants of *B. bassiana* against *Spodoptera litura* [35] have shown an effect on insect mortality.

In the present work, we propose the use of enzymatic cocktails as adjuvants that can increase the action of microbial biological control agents on pest insects. First, a screening study was carried out on isolates of the genera *Beauveria, Trichoderma,* and *Metarhizium* to produce lytic enzymes (chitinases, lipases, and proteases) that can increase the insecticidal action of *B. bassiana* conidia against *D. saccharalis* larvae. It was found that the isolates of *Beauveria bassiana* exhibited lower enzymatic activities than the isolates of the genera *Trichoderma* and *Metarhizium*, which contrasts with a previous work that reported higher enzymatic activity of *Beauveria* strains compared to *Metarhizium* [13, 25]. Also, *Metarhizium* strains co-secreted chitinases and proteases, while two *Trichoderma* strains co-secreted chitinases, lipases, and proteases. It is important to highlight that the culture medium used here were not optimized to produce a specific enzyme group; however, it allowed the selection of those strains with enzymatic potential that can be optimized to produce virulence factors of interest in biological control of agricultural pests.

Entomopathogenic fungi must pass first through the outermost layer, the epicuticle, rich in proteins and lipids, and the procuticle, rich in chitin [36]. The secretion of lipases by entomopathogenic fungi is essential for the degradation of the cuticle and contributes to its virulence and pathogenicity [25]. Fungi are considered the best source to produce lipases with advantages in ease of fermentation and low-cost extraction methods [37]. In this research, lipases were detected at low levels in all tested isolates, except for the isolate *T. harzianum* Th180, and it was the lowest enzymatic activity in the co-production of enzymes in submerged fermentation.

The proteases of the entomopathogens act on the epicuticle helping to weaken this insect barrier before the action carried out by the chitinases [38]. Different types of proteases related to this action have been described, among the most notable are those of the Pr1 family or subtilisin-like serine protease. Conserved Pr1 proteases were shown to function in cuticle degradation, five of them (Pr1C, Pr1G, Pr1A2, Pr1B1 y Pr1B2) individually contributing 19–29% to virulence of *B. bassiana* [39]. The results obtained in this work showed that *M. robertsii* Mt015 isolate secreted the highest levels of protease, followed by *M. brunneum* and *M. anisopliae* isolates. A common feature of these three species is their generalist nature, being able to parasitize over seven arthropod orders [40]. According to previous studies, protease expression levels of *Metarhizium* change with hosts, suggesting a mechanism of interaction of fungal pathogens and their arthropod hosts through differential selective pressures on virulence factors [41]. Thus, the higher capacity of protease production could be correlated with a wide range of hosts.

Besides, chitinases are part of the most important enzymes necessary to hydrolyze the cuticle of insects by entomopathogens, thus, several studies to improve production levels have been done [42, 43]. For example, maximal chitinase production by *Trichoderma viride* was obtained using maltose-modified colloidal chitin as carbon source, at pH 6.5, 35 °C for 96 h incubation [44]. In the present work, the *Trichoderma harzianum* isolate presented the highest levels of chitinase and lipase production, making it a potential candidate for optimizing enzyme production under different fermentation conditions.

The coproduction of several enzymes that act as virulence factors against insects in the same fermentation system would reduce the production cost of enzyme-based biopesticides and would help to drive the adoption of them following the current trends [6, 45]. Thus, this study looked for strains able to coproduce lytic enzymes, and found that *T. harzianum* Th180 was able to coproduce chitinase, lipase, and proteases; and *M. robertsii* Mt015 co-produced proteases and chitinases. There are several works based on the search for enzymatic co-production under different conditions. For example, optimization of solid fermentation conditions of *B. bassiana* showed increased production of exocellulase (10%), endocellulase (63%), chitinase (60%), and *β*-1,3-glucanase (61%) [45]*.* Also, *B. bassiana* in an ultrasound-assisted SmF fermentation produced chitinase and *β*-1,3 glucanases using a chemically defined culture medium supplemented with broths from agro-industrial residues such as rice and soy bran [46]. Furthermore, *T. harzianum* was previously used to co-produce chitinase and *β*-1,3 glucanase in a SmF fermentation system with agro-industrial residues as substrate [47].

Another important aspect to study is the effect of temperature fluctuations, pH changes, the presence of organic solvents, inhibitors or even other enzymes, which would cause instability in the conformation of the enzyme and possible loss of the activity under storage [48]. It was found that the chitinase activity of *M. robertsii* was strongly affected by 30 days of storage at the three temperatures evaluated (− 80, − 20 and 4 °C) in contrast with *T. harzianum*, which could be related to the high levels of protease activity of *M. robertsii* suggesting a direct lytic action on the enzymes present in the extract. In addition, the protease activity in *M. robertsii* was also affected, being more susceptible to storage at − 20 °C. These results suggest the need to develop enzyme stabilization systems to increase the shelf life and improve the storage conditions that allow their development as bioproducts, either directly applied or as adjuvants to microbial biological control agents.

The application of the extracts from both fungi influenced positively the percentage of germination of *B. bassiana* Bv064 conidia and the length of the germ tube, which could be associated with a higher speed of colonization of the insect cuticle and invasion of hemolymph, increasing the virulence [49, 50]. Also, it is hypothesized that fast spore germination and germ tube growth could be reached by adding lytic enzymes to the conidia solution since the enzymes could help to release important nutritional components [51]. Similar results of the action of lytic enzymes on the germination of conidia *M. rileyi* have been reported [52].

In this work, an enhancing effect of the enzymatic extracts of *M. robertsii (*66.7%) and *T. harzianum* (43.5%) on the efficacy of *B. bassiana* against *D. saccharalis* larvae was observed. This result suggests that there might be a significant influence of the higher concentrations of proteases in the extract of *M. robertsii* over the virulence of *B. bassiana* conidia. Proteases are the first weapons in the fungal arsenal to attack the defense barrier of the insect constituted by the proteins of the epicuticle, exocuticle and endocuticle and thus expose the weakened chitin fibers to the action of chitinases [6]. These enhanced effects of the lytic enzymes on the conidia biocontrol activity were dependent on the concentration of the enzyme, with the increase of the efficacy with the enzymes, thus, treatments with higher lytic enzymatic activities and specifically proteases had higher efficacy. Moreover, the enzymatic extracts did not kill or affect the insects when were applied without the microbial biocontrol agent, suggesting that the pathogenic effect corresponds to the microorganisms but not the enzymes alone.

## Conclusion

The present investigation does suggest that the enhancer activity of enzymatic extracts was not species-specific, where the efficacy of conidia of a different genus than the original extracts was observed. This non-species-specific enhancer activity will allow to improve current biopesticides in the market by adding enzymatic extracts accelerating the development process of more efficacious biopesticides. It was also shown that the effect of the lytic enzymes on the conidia biocontrol activity is dependent on the concentration of the enzymes, thus, treatments with higher lytic enzymatic activities and specifically proteases have higher efficacy. Thus, further research must be conducted to identify the right dosage of lytic enzymes to be added to the conidia´s biopesticide to maximize the enhancer effect, which will help in reducing lethal doses of aforementioned biopesticides, as well as expanding the host range to control more pest, which affects diverse crops. Finally, this work constitutes a contribution to the strategy for the improvement of biological control agents using the virulence factors of fungal strains. Still, to accelerate the development process of this type of biopesticides more work should be done on the formulation and stabilization of the lytic enzymes to have a longer shelf life and adequate storage temperature.

### Supplementary Information

Below is the link to the electronic supplementary material.

Supplementary file1 (DOCX 134 kb)

Supplementary file2 (DOCX 1101 kb)

## Data Availability

The datasets generated during and/or analyzed during the current study are available from the corresponding author on reasonable request.
