# Supplementary material for: Assessment of Fungal Lytic Enzymatic Extracts Produced Under Submerged Fermentation as Enhancers of Entomopathogens’ Biological Activity
Source: Curr Microbiol. 2024 Jun 9;81(7):217. doi: 10.1007/s00284-024-03702-z (PMC11162973; doi:10.1007/s00284-024-03702-z)
Supplement: Supplementary file 1 — Supplementary file1 (DOCX 134 kb) [file 284_2024_3702_MOESM1_ESM.docx]

**
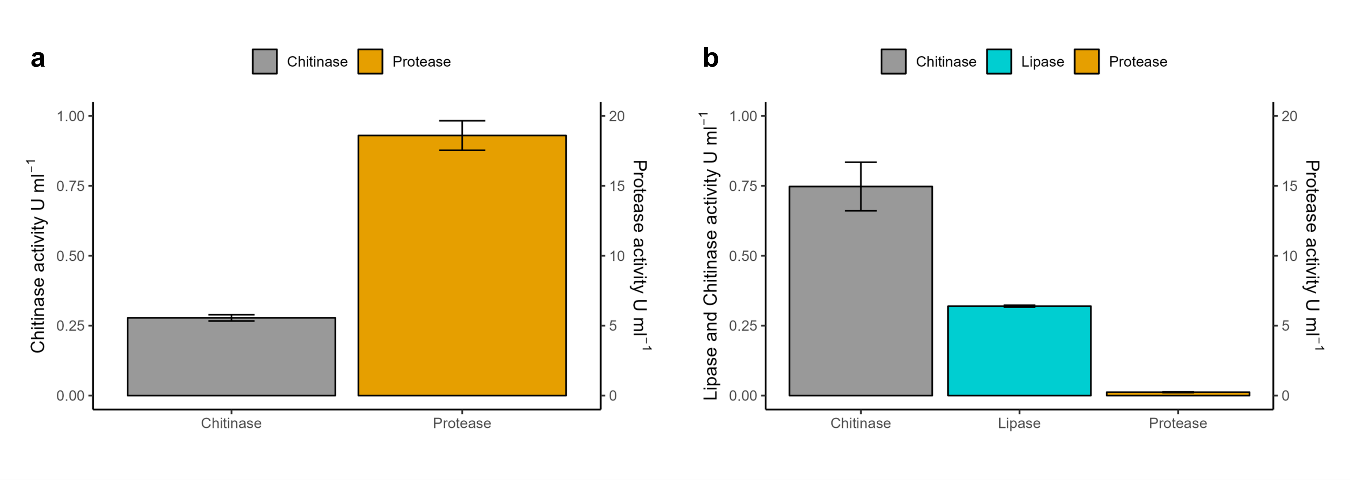
**

**Fig. S1** Concentrated enzymatic extracts (C-ECE) from the SmF process of a) *M. robertsii* Mt015, b) *T. harzianum* Th180.
