# Supplementary material for: Assessment of Fungal Lytic Enzymatic Extracts Produced Under Submerged Fermentation as Enhancers of Entomopathogens’ Biological Activity
Source: Curr Microbiol. 2024 Jun 9;81(7):217. doi: 10.1007/s00284-024-03702-z (PMC11162973; doi:10.1007/s00284-024-03702-z)
Supplement: Supplementary file 2 — Supplementary file2 (DOCX 1101 kb) [file 284_2024_3702_MOESM2_ESM.docx]

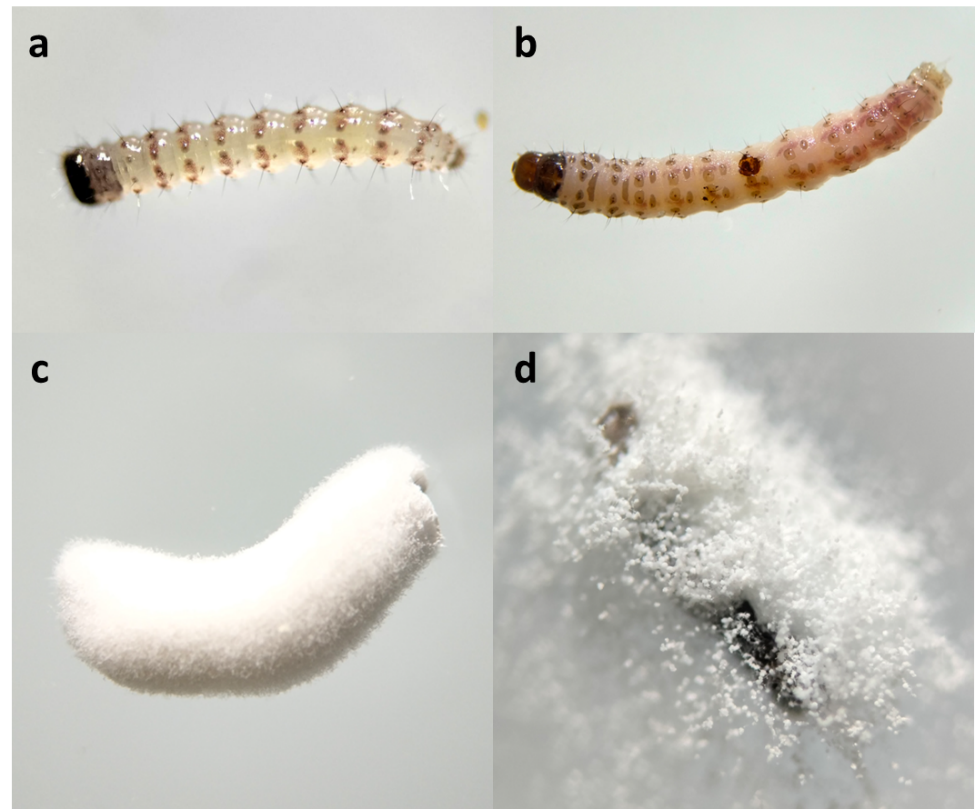


**Fig. S2** Evolution of colonization and sporulation of *B. bassiana* Bv064 on *D. saccharalis* corpse. a) Larva from control treatment, b, c, and d) Larvae from treatments to which conidia of *B. bassiana* Bv064 was applied.
